# Diagnostic Accuracy of 18F-Fluorodeoxyglucose Positron Emission Tomography-Computed Tomography in the Evaluation of Carcinoma of Unknown Primary

**DOI:** 10.4274/mirt.05706

**Published:** 2016-02-10

**Authors:** Saima Riaz, Muhammad Khalid Nawaz, Zia S Faruqui, Syed Ather Saeed Kazmi, Asif Loya, Humayun Bashir

**Affiliations:** 1 Shaukat Khanum Memorial Cancer Hospital and Research Centre, Department of Nuclear Medicine, Lahore, Pakistan; 2 Shaukat Khanum Memorial Cancer Hospital and Research Centre, Department of Radiology, Lahore, Pakistan; 3 Shaukat Khanum Memorial Cancer Hospital and Research Centre, Department of Medical Oncology, Lahore, Pakistan; 4 Shaukat Khanum Memorial Cancer Hospital and Research Centre, Department of Pathology, Lahore, Pakistan

**Keywords:** Carcinoma of unknown primary, 18F-fluorodeoxyglucose, Positron emission tomography, conventional imaging

## Abstract

**Objective:**

Detection of primary tumor site in patients with carcinoma of unknown primary (CUP) syndrome has always been a diagnostic dilemma, necessitating extensive workup. Early detection of primary tumor site coupled with specific therapy improves prognosis. The low detection rate of the primary tumor site can be attributed to the biological behavior or the small size of the primary tumor to be detected by conventional imaging. The objective of this study was to evaluate the diagnostic accuracy of ^18^F-fluorodeoxyglucose (^18^F-FDG) positron emission tomography-computed tomography (PET-CT) in detecting CUP.

**Methods:**

A retrospective, cross-sectional analysis of 100 PET-CT scans of patients with CUP syndrome between November 2009 and December 2013 was performed. Eighteen patients whose final histopathology results could not be obtained for correlation were excluded from analysis. The hypermetabolic sites were assessed in correlation with histopathology. The diagnostic accuracy, sensitivity, specificity, positive predictive value and negative predictive values were assessed for PET-CT.

**Results:**

Out of the 82 patients, primary tumor was correctly identified in 57.3% patients by ^18^F-FDG PET-CT (true positive). The PET-CT scan results were negative for primary site localization in 15% of patients (false negative). While 21% had true negative results, 7.3% displayed false positive results. PET-CT scan upstaged the disease in 27% cases. Overall, the diagnostic accuracy was found to be 78%, sensitivity 80%, specificity 74%, positive predictive value 88.7% and negative predictive value 59%.

**Conclusion:**

Our data supports the utility of ^18^F-FDG PET-CT scan in the localization and staging of CUP syndrome.

## Abstract (tr)

**Amaç:**

Primeri bilinmeyen karsinom (PBK) sendromu olan hastalarda primer tümör yerleşiminin bulunması tanısal bir zorluk oluşturmuş ve detaylı incelemeyi gerektirmiştir. Primer tümör yerleşiminin bulunması ile spesifik tedavinin birleşimi prognozu iyileştirir. Primer tümör yerinin bulunmasındaki düşük oranlar primer tümörün biyolojik davranışına veya konvansiyonel görüntüleme ile saptanamayacak kadar küçük çapta olmasına bağlanabilir. Bu çalışmanın amacı PBK’nın değerlendirilmesinde F-18 florodeoksiglükoz (^18^F-FDG) pozitron emisyon tomografisi/bilgisayarlı tomografinin (PET-BT) tanısal doğruluğunu değerlendirmektir.

**Yöntem:**

Kasım 2009 ve Aralık 2013 tarihleri arasında PBK nedeniyle PET-BT uygulanmış 100 hasta retrospektif, kesitsel olarak analiz edildi. Histopatolojik değerlendirme sonuçlarına ulaşılamayan 18 hasta çalışma dışı bırakıldı. Hipermetabolik alanlar histopatoloji ile karşılaştırıldı. PET-BT için tanısal doğruluk, duyarlılık, özgüllük, pozitif öngörü değeri ve negatif öngörü değeri belirlendi.

**Bulgular:**

Seksen iki hastadan %57,3’ünde primer tümör ^18^F-FDG PET-BT ile doğru olarak tespit edildi (gerçek pozitif). PET-BT sonuçları primer tümör lokalizasyonunu belirlemede %15 hastada negatif idi (yanlış negatif). Hastaların %21’inde gerçek negatif sonuç, %7,3’ünde yanlış pozitif sonuç saptandı. PET-BT ile %27 hastada evre daha ileri olarak bulundu. Genel olarak, tanısal doğruluk %78, duyarlılık %80, özgüllük %74, pozitif öngörü değeri %88,7 ve negatif öngörü değeri %59 olarak saptandı.

**Sonuç:**

Sonuçlarımız ^18^F-FDG PET-BT görüntülemenin PBK sendromunda lokalizasyon ve evreleme amacıyla kullanılabilirliğini desteklemektedir.

## INTRODUCTION

Cancer of unknown or occult primary (CUP) is defined as the detection of metastatic cancer where the site of primary origin remains obscured even after standardized diagnostic work-up. CUPs account for 3 to 5% of all malignancies, and the median age at presentation is 60 years (1,2). CUP is a heterogeneous group of malignancies with variable histology (3). The low rate of primary site detection can be attributed to multiple factors. Detection of a small sized primary lesion is usually challenging on conventional cross-sectional imaging, especially when the lesion is located in anatomically complicated areas such as the head and neck or abdominopelvic regions (4).

The median survival in CUP syndrome has been reported as 10-12 months. The prognosis mainly depends on early detection of the primary tumor site coupled with tumor specific therapy. In case of unidentified primary, empiric chemotherapy regimens are offered (5). Detection of the unknown primary tumor is of crucial importance within this context. Identification of the primary tumor may offer the possibility of a more specific and effective treatment with a consequent improvement in survival.

Metabolic changes are known to precede morphologic changes. Hybrid positron emission tomography-computed tomography (PET-CT) imaging allows whole body survey of metabolic processes together with anatomical correlation, making 18F-fluorodeoxyglucose (^18^F-FDG) PET-CT a useful tool in the search of a primary malignant focus. The key role of whole body ^18^F-FDG PET-CT scan in this field has been shown by previous studies (6,7). Our study was conducted with an aim to assess the frequency of primary sites detected on FDG PET-CT imaging, and its impact on management in our Oncological referrals.

## MATERIALS AND METHODS

This is a retrospective, descriptive, cross-sectional survey of patients with CUP syndrome who were referred to ^18^F-FDG PET-CT scan during the period of November 2009 to December 2013. An approval from the institutional review board was obtained.

### Study Sample

One hundred patients with CUP syndrome fulfilling the inclusion criteria were enrolled in the study including 56 males and 44 females (average age of 54.3 years±14.3 standard deviation (SD)). Seventy-four of 100 patients had histopathologically proven metastatic disease with unknown primary site. Twenty-six out of 100 patients were enrolled with a clinical suspicion of malignancy due to history of profound weight loss or progressive weakness with elevated tumor markers or suspicious lesions on cross-sectional radiological imaging where biopsy was not possible.

Eighteen patients whose histopathology results could not be obtained for correlation after PET-CT were excluded from final analysis.

### Fluorodeoxyglucose Positron Emission Tomography-Computed Tomography Scan Acquisition

All patients underwent ^18^F-FDG PET-CT scan according to the standard protocol. Each patient fasted for 6 hours. A dose of 300 MBq (0.21 mCi/kg body weight) of ^18^F-FDG was injected intravenously to each patient under proper glycemic control. At 60 minutes, whole body PET-CT scan acquisition was performed by a dedicated PET scanner (Phillips Gemini TOF) with 3 min acquisition for each 8-9 bed positions. Contrast enhanced CT scan was acquired over 1 min with a voltage of 70-140 kVp and tube current 80 mA. The CT scan was used for anatomical localization and attenuation correction of PET emission data.

### Image Analysis

The attenuation corrected and uncorrected PET images were evaluated for visual assessment of areas with high metabolic activity. The hypermetabolic foci were anatomically localized on corresponding CT images. Lesion activity was quantified with a Standardized uptake value (SUV). The patient’s history, distribution of pathological lesions and pattern of spread of different tumors was taken into consideration.

### Data Analysis

In the investigation of a primary tumor, detection of the primary malignancy site was considered to be positive (true positive) only when confirmed by histopathology. If findings on PET-CT scan did not turn out to be a primary site by histopathology, these were accepted as ‘false positive’. Unremarkable PET-CT scans were considered as true negative, while false negative results indicated PET-CT scans where the primary site remained obscured.

### Statistical Analysis

The data obtained after quantification was statistically analyzed. Quantitative variables (e.g. age) were presented as mean±SD. Qualitative variables (e.g. gender, identified unknown primary tumors on PET-CT) were expressed as frequency and percentages. The sensitivity, specificity, accuracy, positive predictive value and negative predictive values were calculated by a 2x2 contingency table.

## RESULTS

### Patient Characteristics

Out of the 100 enrolled patients, 74 had histopathologically proven metastatic disease with an unknown primary site. The lymph nodes were the most frequent site of metastasis in 27 patients (36.5%). Liver biopsy was obtained in 15 (20%) patients. The details of metastatic site and histopathologic tumor types are listed in the Table 1.

Twenty-six out of 100 patients were enrolled due to a clinical suspicion of malignancy. Ten (38.5%) out of these 26 patients had hypodense lesions in the liver and 3 (11.5%) had pulmonary nodules on CT, that were all suspicious for metastases but were not biopsied. Likewise one (3.8%) patient had a hypodense lesion in the spleen, 1 (3.8%) had heterogeneous density in the adrenal gland, and 1 (3.8%) had a bulky pancreatic head. Suspicious lesions were detected on magnetic resonance imaging (MRI) scan in the brain in 2 (7.7%) cases, and in the bone in 1 (3.8%) patient. Seven (26.9%) patients had a history of weight loss and progressive weakness along with elevated tumor markers (Table 1).

### Results of Fluorodeoxyglucose Positron Emission Tomography-Computed Tomography Scan

Out of the 100 patients enrolled with CUP syndrome, 18 patients were excluded from final analysis due to lack of information about their final histopathology results or clinical outcome.

Hypermetabolic lesions indicating the primary tumor sites were correctly identified in 47 (57.3%) (true positive). Forty-five of these 47 primary sites were subsequently proven by histopathology. Two scans revealed positive findings in the vocal cords and pyriform sinus, which could not be biopsied; however, these sites were verified as primary sites by MRI scan.

In 6 (7.3%) patients, the hypermetabolic lesions that were identified on PET-CT scans did not turn out to be malignant/primary on subsequent biopsy (false positive). Four of these FDG-avid lesions turned out to be metastatic lesions on biopsy instead of the primary site. In the remaining 2 cases, biopsy of the sigmoid colon showed a tubular adenoma while the biopsy of the FDG -avid renal lesion turned out to be negative for malignancy.

No primary site could be identified in 29 (35%) patients. Within these, 17 (21%) scans were true negatives with unremarkable PET-CT scans without any hypermetabolic site. The remaining 12 (15%) were false negative studies, in which either the known sites of metastasis or additional sites of metastasis showed FDG uptake while primary site could not be identified.

The average SUV_max_ value of the hypermetabolic lesions was reported to be 8.2±4.7 SD.

FDG PET-CT scan led to a shift in disease stage in 38% of the cases; up-staged 27%, while down-staging 11%. Overall, the diagnostic accuracy was found to be 78%, sensitivity 80%, specificity 74%, positive predictive value 88.7% and negative predictive value 59%.

### Histopathology Features of Primary Lesions Identified on Positron Emission Tomography-Computed Tomography Scan

**Figure 1:**
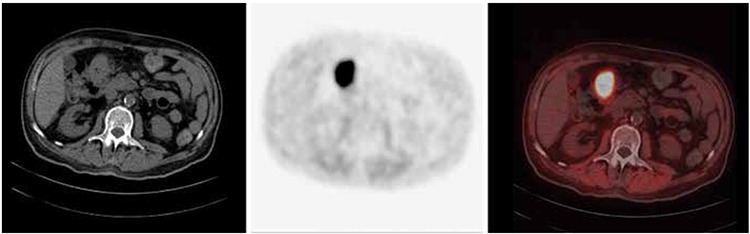
A 57-year-old-male patient with a diagnosis of metastatic adenocarcinoma on liver biopsy with unknown primary. Axial positron emission tomography-computed tomography and fusion images demonstrate hypermetabolic thickening of the transverse colon (SUVmax: 14.6). Biopsy of the transverse colon revealed adenocarcinoma

**Figure 2:**
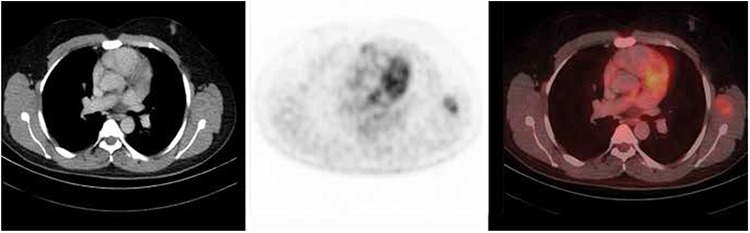
A 46-year-old-male patient with a diagnosis of myelofibrosis on bone marrow biopsy. Axial positron emission tomography-computed tomography and fusion images show hypermetabolic left subscapular soft tissue mass (SUVmax: 3.1). Biopsy of the subscapular mass revealed Non-Hodgkin’s lymphoma

**Figure 3:**
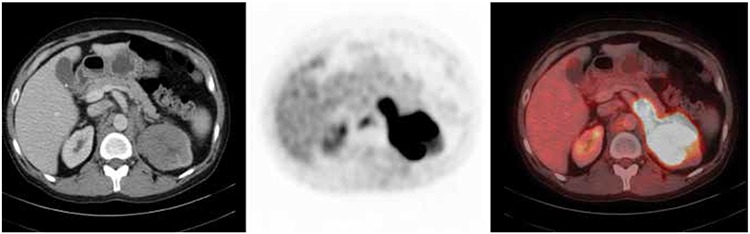
A 43-year-old-male patient with a diagnosis of metastatic neuroendocrine tumor on cervical node biopsy. Axial positron emission tomography-computed tomography and fusion images demonstrate hypermetabolic left renal soft tissue mass (SUVmax: 13.9). The biopsy of this renal mass revealed a neuroendocrine tumor

The identified primary tumor sites were; the lung 10/47 (21.3%), head and neck 10/47 (21.3%), colon and rectum 5/47 (10.6%) (Figure 1), stomach 4/47 (8.5%), liver 2/47 (4.2%), breast 3/47 (6.3%), ovary/adnexa 3/47 (6.3%), pancreas 2/47 (4.2%), small intestine 1/47 (2.1%), mediastinum 2/47 (4.2%), axillary node 1/47 (2.1%), gallbladder 1/47 (2.1%), muscle 1/47 (2.1%) (Figure 2) and kidney 1/47 (2.1%) (Figure 3).

**Figure 4:**
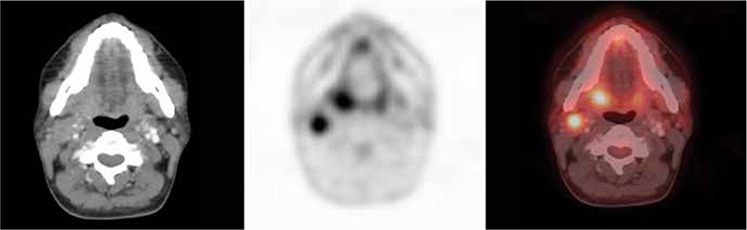
A 39-year-old-female patient with a diagnosis of metastatic squamous cell carcinoma on cervical node biopsy. Axial positron emission tomography-computed tomography and fusion images display hypermetabolic right level II cervical node (SUVmax: 5.9) and right tonsillar soft tissue mass (SUVmax: 6.5) which turned out to be tonsillar squamous cell carcinoma

Out of the 47 detected primary tumors, 45 were further confirmed by histopathology. Thirteen (27.6%) were adenocarcinoma (Gastric adenocarcinoma 4 (30.8%), colorectal adenocarcinoma 5 (38.5%), pancreatic adenocarcinoma 2 (15.4%), breast adenocarcinoma 2 (15.4%)), 10 (21.3%) non-small cell lung cancer, 5 (10.6%) squamous cell carcinoma (Head and neck SCCa 4 (80%) (Figure 4), and mediastinal SCCa 1 (20%)), 3 (6.3%) ovarian carcinoma, and 3 (6.3%) Non-Hodgkin’s lymphoma. Adenoid cystic Ca, nasopharyngeal Ca, follicular thyroid Ca, hepatocellular carcinoma, cholangiocarcinoma, breast invasive ductal carcinoma, alveolar soft part sarcoma, Hodgkin’s disease, renal neuroendocrine tumor and gallbladder Ca were identified in 1 (2.1%) patient each. In two cases, hyper-metabolic lesions were identified in the vocal cord and pyriform sinus, where malignant morphological features were confirmed on subsequent MRI scan.

The primary sites identified on PET-CT scan and the histologic types on subsequent biopsy are presented in Table 2.

## DISCUSSION

Investigating a primary tumor in CUP syndrome has always been a diagnostic challenge. A battery of laboratory and radiological investigations are being used to verify that the primary tumor is unidentifiable. ^18^F-FDG PET-CT is a functional imaging method based on the increased glucose metabolism of malignant foci. Its key role has already been established in the diagnostic workup of a variety of primary tumors (8). In CUP, diagnostic ^18^F-FDG PET-CT is a useful tool for the delineation of a primary focus as it provides functional and morphological information in one step with sets of CT, PET and fused images (9,10,11,12,13). Our study was conducted to evaluate the significance of FDG PET-CT in CUP syndrome in our oncologic referrals. This is the first of its kind data from our country where malignancies tend to present late due to socioeconomic conditions.

The incidence of CUP is strongly related to age, with the highest incidence rate in older men and women (14). A similar statistical data was found in our sample (average age of 54.3 years±14.3 SD), where the majority were in the age group of 50-70 years.

In our study, hybrid FDG PET-CT helped to identify the primary tumor in 57.3% cases. This detection rate is in keeping with the studies in the literature that reported rates between 22-73% (15,16). In our cohort; lung, head and neck, and gastrointestinal tract malignancies were represented at equal frequencies (21.3%). This is also in line with meta-analyses in the literature (17,18). Small lesions in the head and neck, and the abdomen are difficult to localize as these are anatomically complicated regions. Nevertheless, diligent identification of abnormal focal or asymmetric uptake, along with abnormal metabolic process reveal the anatomical localization (4).

In our cohort, adenocarcinoma was the most frequently detected primary tumor (27.6%) followed by non-small cell lung cancer (21.3%), and squamous cell carcinoma (10.6%). In addition, we were able to localize ovarian carcinoma, adenoid cystic Ca, nasopharyngeal Ca, follicular thyroid Ca, hepatocellular carcinoma, cholangiocarcinoma, breast invasive ductal carcinoma, alveolar soft part sarcoma, Hodgkin’s disease, Non-Hodgkin’s lymphoma, renal neuroendocrine tumor and gallbladder Ca. These diverse findings are attributed to different biologic and metabolic behaviors of the histological subtypes of CUP (6). This finding is also a reflection of our status as a tertiary care cancer centre.

High SUV_max_ and FDG uptake pattern on PET-CT scan were found to be important predictors in localizing the primary site of malignancy. The SUV-based quantitative analysis of the hypermetabolic lesions by 18F-FDG PET-CT is the most important diagnostic criteria to distinguish benign from malignant tumors. Currently, a maximum SUV of 2.5 is a widely accepted standard threshold in the diagnosis of malignancy (19). In our study, the average SUV value was reported to be 8.2±4.7 SD. Algin E et al., (20) in a first of its kind study, observed a median SUV_max_ of 8.6 with FDG PET/CT in liver metastases from adenocarcinoma of unknown primary origin (ACUP). They concluded that SUV_max_ is a prognostic factor that influences survival in ACUP. Impact of SUV_max_ on survival was beyond the scope of our current study; however, it is an intriguing area and should be studied across the heterogeneous spectrum of CUP syndrome to identify favorable factors.

FDG PET-CT could not localize primary tumor site in 35% of patients. Despite the high rate of detection of the primary tumor site by FDG PET-CT, certain types of tumors remain obscured. As reported by Alberini JL et al., (21) elevated glucose consumption with high FDG uptake is detected in high-grade epithelial tumors and vice versa. Low-grade tumors with low FDG uptake are barely delineated and remain hidden. Furthermore, FDG PET-CT scan can only help in the mapping of macroscopic disease, as it is impossible to localize microscopic disease in spite of the technique’s high resolution. Other hypothesis for obscured primary sites is immune-mediated destruction or spontaneous regression (22).

False positive results were detected only in 6 (15%) patients, where the hypermetabolic lesions identified on PET-CT scans did not turn out to be malignant/primary on subsequent biopsy. These false positive findings were observed in the abdomino-pelvic region, where physiologic tracer uptake usually mimics pathologic lesions (4).

In our study, disease stage was modified in 38% of the cases. This is slightly better than the 34.7% stated in a recent review by Pawaskar and Basu (22). Modification in treatment regimen and impact on survival was beyond the scope of our current study. However, in the literature, it has been reported that the survival rate of patients with hypermetabolic lesions is significantly lower (22,23).

One limitation to this study was the relatively smaller number of cases, which is attributed to the extremely heterogeneous cohort of patients with CUP syndrome. Basing conclusions on results from a small group is a limitation to proving efficacy of FDG PET-CT scan in the diagnosis of unknown primary tumor site; however, the results suggest that incorporation of FDG PET-CT scan in the early diagnostic phase can improve management.

In spite of the heterogeneity of CUP syndrome, the scientific literature and the findings of our study agree to the concept that FDG PET-CT scan is a useful diagnostic tool in detecting an unidentified primary tumor site. Currently, the role of PET-CT scan has been defined in the staging of cancer patients in Oncology (24). The indications for FDG PET-CT for CUP include primary tumor site localization and disease staging. In addition to 18F-FDG, the upcoming PET radiopharmaceuticals, which map protein synthesis and various receptors in tumors, are also being evaluated for use in localizing obscured primary malignant lesions, though with limited success (25).

## CONCLUSION

Our study is in conformity with the published literature on the role of hybrid ^18^F-FDG PET-CT imaging in localization of unknown primary tumors across all body regions, as well as in staging CUP syndrome.

### Compliance with Ethical Standards

The study complies with the ethical standards. Approval was taken from the Institutional review board (IRB). As per IRB, no more than the nominal risk is contained since the information of human subjects is documented without any identifiers as well as presenting an entirely anonymous research data set (IRB approval letter can be provided if required).

## Figures and Tables

**Table 1:**
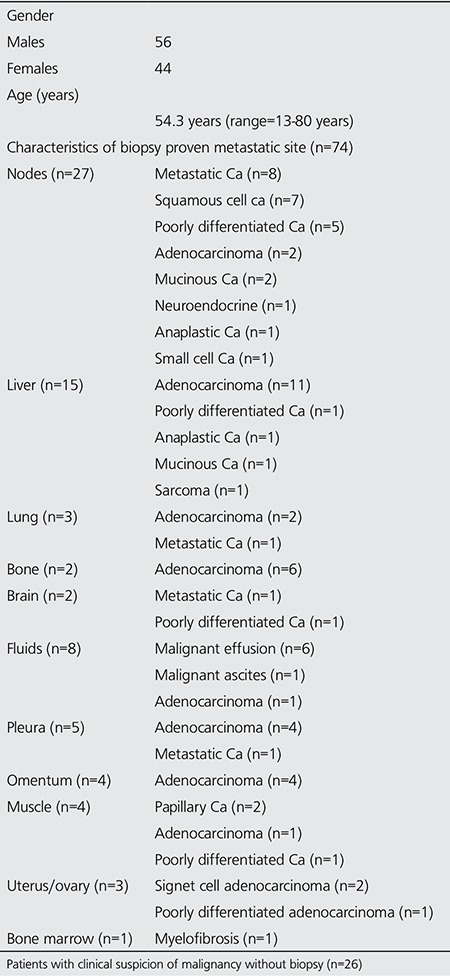
Patient and disease characteristics

**Table 2:**
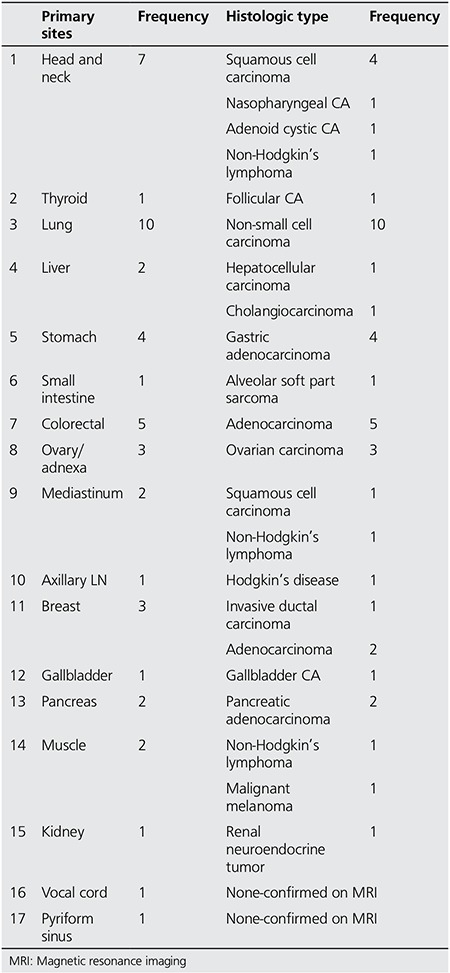
Frequencies of primary sites identified on positron emission tomography-computed tomography scan and histologic types (n=47/100)
